# An expanded variant list and assembly annotation identifies multiple novel coding and noncoding genes for prostate cancer risk using a normal prostate tissue eQTL data set

**DOI:** 10.1371/journal.pone.0214588

**Published:** 2019-04-08

**Authors:** Melissa S. DeRycke, Melissa C. Larson, Asha A. Nair, Shannon K. McDonnell, Amy J. French, Lori S. Tillmans, Shaun M. Riska, Saurabh Baheti, Zachary C. Fogarty, Nicholas B. Larson, Daniel R. O’Brien, John C. Cheville, Liang Wang, Daniel J. Schaid, Stephen N. Thibodeau

**Affiliations:** 1 Department of Laboratory Medicine and Pathology, Mayo Clinic College of Medicine, SW, Rochester, Minnesota, United States of America; 2 Department of Health Sciences Research, Mayo Clinic College of Medicine, SW, Rochester, Minnesota, United States of America; 3 Department of Pathology, Medical College of Wisconsin, Milwaukee, Wisconsin, United States of America; University of South Alabama Mitchell Cancer Institute, UNITED STATES

## Abstract

Prostate cancer (PrCa) is highly heritable; 284 variants have been identified to date that are associated with increased prostate cancer risk, yet few genes contributing to its development are known. Expression quantitative trait loci (eQTL) studies link variants with affected genes, helping to determine how these variants might regulate gene expression and may influence prostate cancer risk. In the current study, we performed eQTL analysis on 471 normal prostate epithelium samples and 249 PrCa-risk variants in 196 risk loci, utilizing RNA sequencing transcriptome data based on ENSEMBL gene definition and genome-wide variant data. We identified a total of 213 genes associated with known PrCa-risk variants, including 141 protein-coding genes, 16 lncRNAs, and 56 other non-coding RNA species with differential expression. Compared to our previous analysis, where RefSeq was used for gene annotation, we identified an additional 130 expressed genes associated with known PrCa-risk variants. We detected an eQTL signal for more than half (n = 102, 52%) of the 196 loci tested; 52 (51%) of which were a Group 1 signal, indicating high linkage disequilibrium (LD) between the peak eQTL variant and the PrCa-risk variant (r^2^>0.5) and may help explain how risk variants influence the development of prostate cancer.

## Introduction

Prostate cancer (PrCa) is the most commonly diagnosed and second most deadly cancer in men in the US, with over 160,000 estimated new cases and over 29,000 deaths in 2018.[1] There is a substantial genetic component to risk of PrCa, with heritability estimates as high as 58%.[2] Variants in several genes have been associated with increased risk, including *HOXB13*, *BRCA2*, *BRCA1*, *ATM*, *CHEK2*, and the mismatch repair genes; however, these occur in only a small fraction of cases and explaining the remaining heritability of PrCa remains elusive.[3–11]

Several genome-wide association studies (GWASs) have investigated the genetics of PrCa risk, identifying 284 variant associations.[12–30] Many of the identified variants reside in non-coding regions of the genome, making biological interpretation difficult. Additionally, many of these variants are likely tag-SNPs that are in linkage disequilibrium (LD) with the causal variant conferring increased predisposition to PrCa.

Expression quantitative trait locus (eQTL) analyses can be useful for identifying candidate causal genes by linking risk SNPs to changes in gene expression. Variants influencing expression often lie in regulatory regions, such as enhancers or promoters, which may not be physically close to the gene they impact. PrCa-risk variants may also affect the expression of non-coding RNAs (ncRNAs), such as long non-coding RNA (lncRNA), miRNA, and pseudogenes. lncRNAs and other ncRNA may impact PrCa risk by altering expression of protein-coding genes through a variety of mechanisms, including binding to chromatin, proteins, or other RNAs and acting either as a scaffold or to sequester other functional RNAs.[31]

Results of eQTL studies can vary greatly depending on the genes reference assembly used in the analysis. RefSeq is a highly curated source consisting of approximately 24,000 genes.[32] In contrast, ENSEMBL gene annotation has over 57,000 genes and also includes numerous ncRNAs. Using a more comprehensive gene annotation such as ENSEMBL allows more reads to be mapped during analysis and may help identify additional PrCa-risk eQTL signals not found using more conservative reference assemblies.

Previously, we completed an eQTL study consisting of normal prostate epithelial cells in 471 samples and identified 88 genes whose expression was associated with PrCa-risk variants.[33] However, there remained a high number of PrCa-risk variants (n = 87, 60%) for which a target gene was not identified. In this study, we have expanded the scope of our investigation by including an additional 135 PrCa-risk variants and by using ENSEMBL gene annotation, rather than RefSeq, to characterize gene abundance. In total, we investigated 249 PrCa-risk variants in 196 loci of interest that collectively harbored 3,074 protein coding genes, 348 lncRNA, and 1,289 other RNA species.

## Results

### PrCa-risk variant- and gene-based eQTL

After quality control, 471 samples of prostate epithelium underwent RNA sequencing and genotyping. PrCa-risk variants (n = 284) were chosen based on the literature review and included 135 newly published variants not evaluated in our previous study (**S1 Table**).[33] After review, 35 of the 284 variants were excluded due to low MAF, being in perfect LD with another PrCa-risk variant, or not being present in the observed or imputed genotype file (**S1 Table**), leaving 249 for the primary analysis. PrCa-risk variants were grouped into 196 risk loci. Forty-five risk loci corresponded to multiple PrCa-risk variants in LD: 38 loci had two, six loci had three, and one locus had four risk variants.

**Fig 1:**
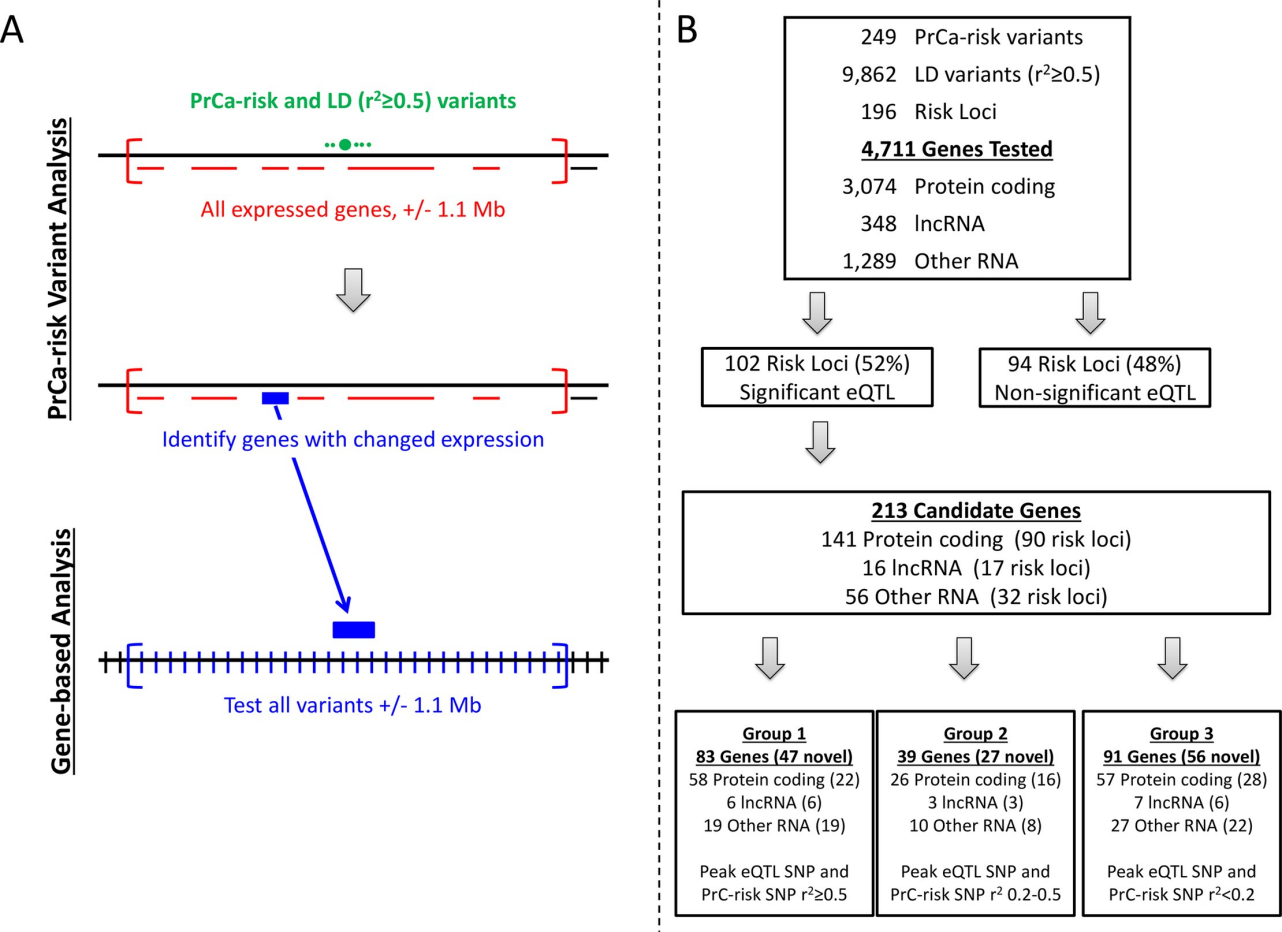
Study Design. **(A) Flow diagram of stages of analyses.** In the variant-based analysis, PrCa-risk variant(s) (green bar) and all nearby variants in LD were tested for eQTL signals against all genes +/- 1.1 Mb (red lines) to identify target genes (blue line). In the gene-based analysis, all variants (blue bars) +/- 1.1 Mb from an identified gene (blue line) were evaluated for eQTL associations. **(B) Numbers of variants and genes identified during analyses.** A total of 10,111 variants (249 PrCa-risk and 9,613 LD variants) in 196 risk loci were tested in the first stage of analysis. Significant eQTL signals were found in 213 genes in 102 loci. Significant genes were identified as protein coding, lncRNA, or other RNA and then grouped according to the LD between the peak eQTL variant and the PrCa-risk variant. Numbers in the Groups indicate the total number of unique genes identified (number of unique novel genes identified. i.e., not previously reported in Thibodeau et al. 2016). Several genes fell into multiple groups due to the gene’s proximity to multiple risk loci. In this figure, each gene is counted only once and is included in the group with the highest LD with the PrCa-risk SNP. For a full list of all genes, please see S2 Table.

In Stage 1, we analyzed 9,862 SNPs (PrCa-risk and LD variants) and 4,711 genes, which included protein coding genes (n = 3,074), lncRNA (n = 348), and other RNA species (n = 1,289), for a total of 306,365 association tests (**Fig 1A**, **S2 Table**). Of the 196 risk loci, 102 (52.0%) had at least one gene with a significant eQTL signal. A total of 213 unique genes were identified with significant eQTL signals, including 141 protein coding genes, 16 lncRNA, and 56 other RNA species (**Fig 1B**).

Genes associated with a significant eQTL signal were considered targets for PrCa risk. In Stage 2 of the analysis, each of these target genes was further evaluated by analyzing all nearby (+/- 1.1 Mb from gene transcription start and end) variants to determine their influence on expression. A total of 308,262 tests were conducted (9,915 variants and 213 genes) in this stage.

It is likely that some significant eQTL signals will reflect normal, prostate tissue-specific signals. To aid interpretation and focus on genes of probable relevance to PrCa, significant target genes were classified according to the LD between the original PrCa-risk variant and the variant exhibiting the strongest eQTL association (minimum p-value) for that gene. The latter peak eQTL variant was defined considering all variants analyzed in both the primary- and second-stage. Genes with high LD (r^2^ ≥ 0.5) between the PrCa-risk variant and the peak eQTL variant were considered the most likely to be related to PrCa-risk and classified as Group 1. Those with lower LD between the PrCa-risk variant and the peak eQTL variant (r^2^ in [0.2–0.5) or r^2^ <0.2) were classified as Group 2 and Group 3, respectively.

### Protein coding genes

**Fig 2:**
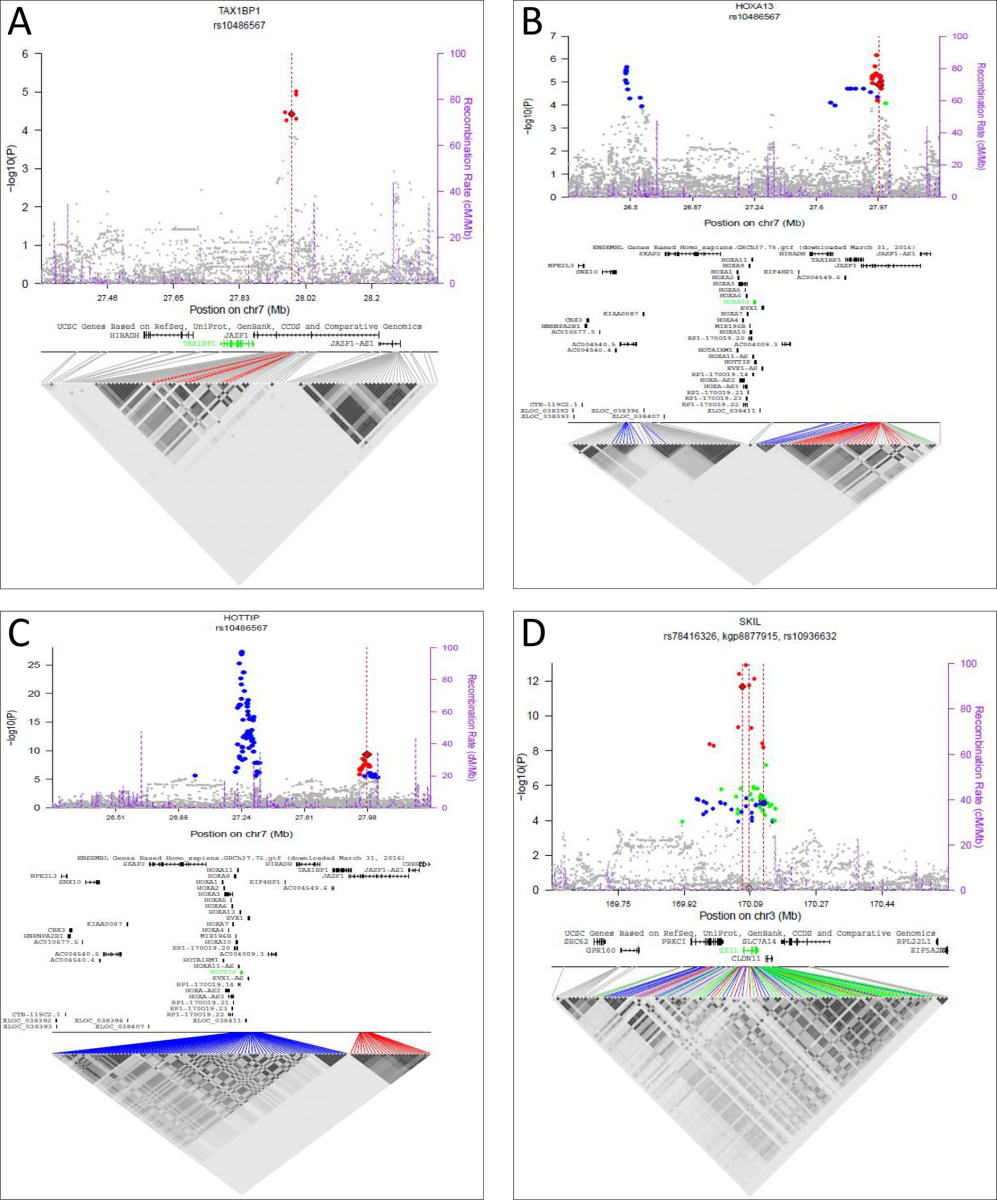
*TAX1BP1*, *HOXA13*, *HOTTIP*, and *SKIL* risk loci. Risk loci are shown for **(A)** *TAX1BP1*, **(B)** *HOXA13*, **(C)** *HOTTIP*, and **(D)** *SKIL*. The *x* axis shows the chromosomal position of the variants (with analyzed gene in the region below) and the *y* axis is the -log10 (P-value) obtained by regressing normalized expression levels for the gene listed in the panel title on the number of minor alleles of each variant genotype adjusted for histologic characteristics and 14 expression principal components. PrCa-risk variant is indicated by the dashed red vertical line and the eQTL variant with a diamond. All significant variants are colored, with variants in high LD with the PrCa-risk variant in red (LD>0.5); those with LD between 0.2–0.5 in green, and those with LD≤0.2 in blue. The right y axis shows the recombination rate (purple dashed lines mark recombination locations). The bottom half of each panel contains an LD heat mat of the significant variants in the region.

In total, 141 protein coding genes in 90 risk loci were identified with significant eQTL signals (**S2 Table**), including 66 genes not found in our previous study.[33] The majority of risk loci with a significant eQTL signal contained a single significant gene (n = 51), while the remaining 39 contained two (n = 20), three (n = 11), four (n = 3), or six or more (n = 5) significant genes. Almost half (n = 58, 41%) of the protein coding genes were categorized as Group 1 (**Fig 1B, Fig 2**), implicating these genes as potential candidates for PrCa-risk. Three previously studied loci contained newly identified Group 1 protein coding genes: locus 81 (chr7:27876563–28076563, *HOXA13* and *TAX1BP1*), locus 84 (chr7:97707882–97916327, *TECPR1*), and locus 178 (chr19:54697848–54897848, *LILRA3*. The remaining newly identified Group 1 protein coding genes were in loci not previously tested.

### Non-coding genes

We identified 16 lncRNAs in 17 risk loci and 56 other RNA genes in 32 risk loci with significant eQTL signals. Over one-third (35%) of the 72 non-coding genes had peak eQTL variants in high LD with PrCa-risk variants, thus were considered Group 1. Twelve risk-loci contained an eQTL signal with a non-coding gene only, which would have gone undetected in our previous analysis. For example, *RP11-351C8*.*1* was a newly identified lncRNA with a Group 1 eQTL signal in locus 88 (chr8:127823720–128206880). Two other loci also had lncRNAs alone associated with significant eQTL signals, including *LINC00303* (locus 5, chr1:204391549–204618842) and *RP11-38L15*.*2* (locus 109, chr10:47446323–47646323); however both peak eQTL variants were in low LD with the PrCa-risk variant (Groups 2 and 3, respectively) and were considered less likely to influence PrCa risk.

Of the 32 loci with a significant eQTL signal in the other RNA category (**Fig 1B**), 11 contained Group 1 eQTL signal(s); six of which had a single Group 1 association, four loci contained two Group 1 associations, and a single locus contained five Group 1 eQTL signals. Of the 11 loci, four contained no additional Group 1 signals with either protein-coding or lncRNA genes (loci 19, 31, 61, and 136). In two of these four loci, no additional eQTL signals of any Group were identified. Locus 19 (chr2:111793096–111993096) was a new locus with a single PrCa-risk SNP (rs11691517), while locus 31 (chr3:87010674–87567332, rs2660753) was tested previously with no eQTL signal identified.

## Discussion

In this study, we identified 213 genes in 102 risk loci with eQTL signals in normal prostate that may be important for the development of PrCa. These included 141 protein coding genes, 16 known lncRNAs and 56 other RNA genes. We identified putative target genes for approximately half of the 196 risk loci tested (102, 52%), 52 (51%) of which contained a Group 1 eQTL signal. Of the 213 genes identified, 130 were considered novel, having not been identified in our previous analysis.[33] While protein coding genes were the most commonly identified of the novel genes (n = 66, 51%), an additional 12% (n = 15) were identified for lncRNAs and 38% (n = 49) were identified for other RNA species putative targets were also found (**Fig 1B**).

We previously completed a similar analysis of 146 PrCa-risk variants using RefSeq gene annotation, identifying 127 risk loci and 88 genes with a significant eQTL signal. In the current study we utilized ENSEMBL for gene annotation. The ENSEMBL annotation has several features that are useful when trying to identify novel genes in transcriptional studies. First, ENSEMBL covers more of the genome, allowing for more transcripts to be successfully mapped. It also contains additional isoforms of genes that are not present in RefSeq, increasing the size and complexity of the annotation. Finally, a large number of non-coding RNAs are present in ENSEMBL that are not present in RefSeq. All of these factors contribute to the increased number of genes identified when using ENSEMBL annotations. Using ENSEMBL, an average of 75.7% of reads mapped to genes compared to 68.8% when using RefSeq gene definition. This allowed us to investigate novel ncRNA that can act as important regulators of gene expression. Using ENSEMBL along with the addition of 69 new risk loci and 135 previously untested PrCa-risk variants, we identified an additional 130 eQTL target genes in 78 risk loci, including 10 loci with newly identified Group 1 eQTL signals in previously negative loci. Thirty-seven of the 78 risk loci were new regions in this study not previously studied. Of the 88 genes originally identified, two were not present in ENSEMBL annotation (*LOC284578* and *LOC284581*). Of the remaining 86 genes present in both our previous and current study, 83 were replicated. The remaining three previously identified target genes were no longer significant in our current analysis (*KRT18*, *SFXN2*, and *TUBA1B*). For *TUBA1B*, the chromosomal start and stop positions changed when using the ENSEMBL annotation, resulting in the change of significance. For *KRT18* and *SFXN2*, both were borderline significant in the first stage of analysis (p = 0.014 and p = 0.020, respectively).

Although there is considerable variation in the definitions of non-coding RNA species, those having >200 nucleotides have been classified as lncRNA. Several further classifications have been proposed, such as intergenic and antisense, however, these have been inconsistently applied. For this study, we excluded lncRNAs that overlapped with any protein coding gene and focused only on intergenic lncRNAs. Thus, it may be that there are additional lncRNAs with significant eQTL signals in prostate tissue that we did not find due to overlap with other genes. Of the 72 non-coding genes identified in the current study, only seven were previously detected (two lncRNAs, *HOTTIP* and *AC011526*.*1*, and five other non-coding genes, *DBIL5P*, *HCG4*, *HLA-L*, *PSORS1C3*, and *RP11-18I14*.*19*). Note that we recognize and describe *HOTTIP* hereafter as a well-known lncRNA, despite having the classification of anti-sense RNA by ENSEMBL. The remaining 65 (90%) non-coding sequences were newly identified.[33]

For 12 loci, a significant eQTL signal was identified for a non-coding gene alone. The paucity of information available for these sequences makes it difficult to tease apart how they may influence the risk of PrCa. However, numerous studies have described several unrelated ncRNAs that are associated with PrCa. *SChLAP1*, a lncRNA, modulates gene expression in PrCa through interactions with the SWI/SNF chromatin remodeling complex and its expression is associated with aggressive disease.[34] *NEAT1*, a lncRNA target of ERα signaling, can help PrCa cells escape androgen ablation therapy by acting as a transcriptional regulator and promoting the expression of PrCa-specific genes. It has also been reported to promote PrCa growth by binding to *SRC3*, an androgen receptor co-activator and inducing increased expression of *IGF1R*, resulting in activation of the AKT signaling pathway and cancer cell growth. [35, 36] Many other ncRNAs have been implicated in PrCa (for a review, see Mitobe et al.). Given the involvement of lncRNA or ncRNA genes in PrCa, the twelve loci identified in the current study are excellent targets for additional research into the complex regulatory networks of ncRNA in PrCa. [37]

In addition to the loci for which a lncRNA was identified alone, we identified several other loci that contained both Group 1 protein coding genes and ncRNAs, such as locus 11 (chr2:20778153–20988265, *GDF7*, *AC012065*.*7*, and *RP11-130L8*.*2*), locus 45 (chr:95405592–95662877, *BMPR1B* and *RP11-554D13*.*1*) and locus 53 (chr5:1791800–1995829, *IRX4*, *CDT-2194D22*.*2*, *CDT-2194D22*.*3*, and *CDT-2194D22*.*4*). Determining if these genes interact or if they are independent signals will be critical to understanding how these loci influence PrCa-risk.

Of the 213 genes identified, forty-two had more than one significant eQTL signal. Most of these genes had two significant signals, while eleven genes had three and two genes (*MICA* and *RNF5*) had four. The multiple significant loci for a single gene may represent multiple different regulatory regions or mechanisms for modulating gene expression or may be due to residual LD between neighboring risk loci (i.e., despite low LD between nearby PrCa-risk variants; variants in high LD with those PrCa-risk variants may also be in high LD leading to multiple ‘signals’ yet a single regulatory region). Careful experiments will be needed to untangle how these signals interact to regulate gene expression.

We identified significant eQTL signals for 66 new protein coding genes not previously identified.[33] In an effort to identify candidate regulatory elements for three of the Group 1 eQTL genes (*TAX1BP1*, *HOXA13*, and *SKIL*), we utilized publically available datasets containing functional annotation. *TAX1BP1* (locus 81, chr7:27876563–28076563; **Fig 2A**) is required to stop NF-κB signaling triggered via the proinflammatory cytokines TNF-α, IL-1, and LPS.[38] Dysregulation of inflammation is a hallmark of many types of cancer and can play a role in the initiation, promotion, and progression of carcinogenesis.[39] Based on its ability to regulate several key genes through NF-kB, including tumor suppressors and those involved in cell survival and proliferation, *TAX1BP1* may contribute to the development of PrCa.[40] Multiple eQTL variants in our current study have features that indicate they may influence the expression of *TAX1BP1*, including their presence in PrCa enhancer regions, their presence in transcription factor binding motifs, or the presence of transcription factor proteins being bound in PrCa cell lines (**S3 Table**).[12, 41, 42]

In the same locus as *TAX1BP1* is *HOXA13* (**Fig 2B**). Interestingly, the lncRNA *HOTTIP* (**Fig 2C**), a Group 3 eQTL signal, is also a significant signal at this locus and it can regulate the expression of distal HOX genes, such as *HOXA13*, through binding *WDR5* and promoting H3K4me3 over the HOXA locus.[43] Expression of *HOTTIP* is increased in several cancer types compared to their normal cellular counterpart, including esophageal, gastric, non-small cell lung, pancreatic, and prostate cancer. In each of these cancers, increased expression of *HOTTIP* is associated with increased expression of *HOXA13 in vitro*, leading to increased proliferation and migration.[44–50] This locus was investigated in our previous study and contained only a single significant eQTL signal, *HOTTIP*.[33] Unsurprisingly, 33% of the top 100 eQTL variants (those with the smallest FDR q-value) were shared between *TAX1BP1* and *HOXA13*, while 16% were shared between both the protein coding genes and the lncRNA *HOTTIP*.

*SKIL* was another novel target gene identified as a Group 3 signal in a previously evaluated locus that did not correspond to any significant findings (locus 41) and in a second locus (locus 42, chr3:169974517–170230102) as a Group 1 signal (**Fig 2D**). *SKIL*, encoding the SKI-like protein (SKIL), plays important roles in TGFβ signaling; depending on the context, it can act as either an oncogene or a tumor suppressor.[51, 52] SKIL functions as a negative regulator of TGF signaling. When no ligand is present, it binds to nuclear SMAD proteins to repress transcription. [53, 54] Upon activation of TGFB signaling, SKIL is ubiquitinated and degraded, allowing transcription to occur.[55] Rearrangements placing *SKIL* near androgen regulated promoters have been described, including *TMPRSS2*, *SLC45A3*, *ACPP*, *MIPOLI*, and *HMGN2P46*. In a study of 540 PrCa cases, 1% had a gene fusion involving *SKIL*, suggesting that regulation of *SKIL* expression is important in PrCa.[56] The 100 eQTL variants with the lowest FDR q-value for *SKIL* are shown in **S4 Table**. SNP rs78416326 is an interesting variant for the eQTL signal for *SKIL*. It is present in the promoter of *SKIL* and lies in predicted binding motifs for three transcription factors, NR2F1, HNF4, and COUP. A second eQTL signal variant for *SKIL*, rs17826519, resides in a PrCa enhancer region and binding region for two transcription factors.

Finally, we investigated the available functional annotation of the top eQTL signals present in the lncRNA *RP11-351C8* (locus 88, chr8:127823720–128206880; **S5 Table**). Three variants of interest reside in the locus, and all three (rs7844107, rs11775448, rs980170) reside in known PrCa enhancers and predicted transcription factor binding motifs.[12, 57] Further studies of the top eQTL variants in the above mentioned genes and lncRNA are warranted to help clarify which one(s) functionally contribute to the change in expression and may influence PrCa-risk.

Two recent studies have also looked for eQTL signals in prostate cancer. In both studies, The Cancer Genome Atlas (TCGA) data from prostate cancer samples was used for the eQTL discovery. Twenty-seven significant eQTL signals were found between the two studies. Al Olama et al. utilized TCGA data from 145 prostate cancer and 45 normal tissue samples to investigate 75 variants in 55 regions and identified 16 significant eQTL signals.[24] We identified 11 of the 16 signals they detected plus an additional 36 eQTL signals in regions studied by both. Yan et al. found 11 significant eQTL signals in 55 regions, utilizing the prostate cancer sample data available through the TCGA. We found eight of the same signals, as well as 26 more in regions present in both. The difference between our findings and these others is likely due to several factors. First, we used normal prostate epithelium for the expression analysis whereas Al Olama et al. and Yan et al. both utilized prostate tumor samples. Second, we included lncRNA and other RNA species in our analyses, where both of the other studies focused on protein coding genes only. During our first stage analysis, we included genes located within a larger region around the original PrCa-risk variant (+/-1.1 Mb) than the other studies (+/-0.5Mb); this, coupled with the larger sample size of our dataset, likely influenced our results to have increased signals detected.

There are several limitations to our study. Ethnicity is a well-established risk factor for PrCa and African American men have the highest risk. Our samples were solely from men of European ancestry, however, preventing us from determining variants and eQTL signals that may be influential in this population. Several PrCa-risk variants were excluded because they were not present in our genotyping data. It is likely that additional eQTL signals were not identified because of this gap; a targeted genotyping panel may help identify missed signals. Additionally, ENSEMBL is both more complex and less conservative genome annotation than RefSeq, which may result in more ambiguous mapping of transcripts. While a more conservative annotation is preferred for studies involving stable RNA expression, a more complex annotation is useful to identify unknown biological mechanisms such as those we searched for. [32] ENSEMBL consists of 196,354 isoforms for 57,773 genes. When several isoforms for a gene exist, ENSEMBL combines all unique exons from such isoforms into a gene definition. We focused on cis-eQTL signals using this aggregated gene definition, preventing us from identifying any trans-eQTLs or rarer isoform-specific eQTLs. Several loci with significant eQTL signals contained only Group 2 or 3 signals; whether these eQTL signals represent true targets of the PrCa-risk variant is unknown. The decreased LD between the risk variant and peak eQTL variant for these genes suggests that these may not be the true risk target. EQTL signals in Group 3 are more likely to represent more general prostate tissue specific eQTLs, while those in Group 2 likely contain both prostate tissue specific as well as PrCa risk specific eQTLs. Taking this into account, the actual number of PrCa-risk variants where we have identified a candidate causal gene is likely to be between 30% (Group 1 genes only, 75 of 249 PrCa-risk variants) and 41% (Groups 1 and 2 genes combined, 101 PrCa-risk variants). Finally, as mentioned above, the consequence of eQTL signals in non-coding RNA is unknown. We identified 12 loci where the only significant eQTL signal was a non-coding gene; understanding how these influence risk will require further study.

In summary, we have identified numerous new eQTL signals present in normal prostate tissue. We have identified genes for 52% of the loci studied; 52 (26%) of the loci contained Group 1 target genes that are likely to play a role in PrCa development. Further functional experiments will be needed to understand how the identified variants and genes influence the development of PrCa risk.

## Materials and methods

### Case selection

Informed consent was obtained from all subjects; the study was approved by the Mayo Clinic Institutional Review Board. Full details on case selection are detailed in Thibodeau, et al.[33] Briefly, normal prostate tissue from patients with either radical prostatectomy or cystoprostatectomy was acquired from an archive collection. After review of the initial surgical hematoxylin and eosin (H&E) slide, 916 cases were identified with no prostate tumor present and a Gleason score ≤7 for the presenting tumor. New H&E slides of these cases were re-examined to select samples with the following characteristics: (1) absence of PrCa; (2) absence of high-grade prostatic intraepithelial neoplasia and benign prostatic hyperplasia; (3) normal prostatic epithelial glands representing ≥40% of all cells; (4) lymphocytic population representing ≤2% of all cells; and (5) the normal epithelium was from the posterior region of the prostate. Of these, 565 cases met the inclusion criteria.

### Tissue processing

DNA was extracted from frozen tissue samples using the Puregene tissue extraction protocol per the manufacturer’s recommendations (Qiagen, Hilden, Germany), as described previously.[33] RNA was extracted using the QIAGEN miRNeasy Mini Kit and the QIAcube instrument in accordance with the manufacturer’s instructions (Qiagen, Hilden, Germany). Upon review of additional H&E stained slides adjacent to the slides used for DNA and RNA extraction and RNA quality, 71 cases were excluded, yielding 494 samples eligible for genome-wide DNA genotyping and RNA sequencing.

### RNA sequencing and pre-processing

Complete methods for RNA sequencing are detailed in Thibodeau, et al.[33] RNA libraries were prepared using the TruSeq RNA Sample Prep Kit v2 (Illumina, San Diego, CA) according to the manufacturer’s instruction. One sample failed library prep and was excluded from the study. Samples were sequenced on an Illumina HiSeq 2000 using TruSeq SBS sequencing kit version 3. A minimum of 50 million total reads per sample was required for analysis; 234 samples with <50 million total reads were re-sequenced and BAM files were merged if no quality issues were identified. RNA-seq data were analyzed using MAP-R-Seq pipeline, an integrated suite of open-source bioinformatics tools, along with in-house developed methods.[58] Gene counts were quantified for 55,601 genes based on ENSEMBL gene annotation. For genes mapping to both chromosomes X and Y, only the chromosome X version was retained.

### RNA-sequencing quality control and normalization

We assessed the existence and functional form of bias as well as the success of expression normalization using graphical methods. This included the influence of flow cell, lane, tissue cut-group, extraction-group, and library prep plate on global mRNA abundance. We excluded genes as insufficiently expressed if they had a median raw gene count < 8, reducing the number of expressed genes (including protein coding, lncRNA, and other RNA species) to 27,576 for the final data analysis. In order to remove potential biases such as GC-content and to account for differences in sequencing depth, the gene counts were normalized using conditional quantile normalization.[59]

### Genotyping

Illumina’s Infinium 2.5M bead array was used to genotype samples based on the manufacturer’s protocol (Illumina, San Diego, CA), as described previously.[33] From a total of 493 genotyped normal prostate tissue samples, 22 were excluded after quality control of the genotypes; 5 due to low call rate (<95%), 10 were found to have a high proportion of African-American ancestry, and 7 had low genotype concordance (<98%) compared with mRNA called variants. The final data set consisted of 471 normal prostate tissue samples (453 low Gleason grade PrCa cases and 18 cystoprostatectomy cases). After quality control exclusions (genotyping failure, duplicate variants, call rate <95%, Hardy-Weinberg equilibrium P-value <1E-5, and minor allele frequency (MAF) <1%), 1,541,368 observed autosomal and chromosome X variants remained. SHAPEIT and IMPUTE2 with reference panels from the 1000 Genomes Phase I integrated variant set were used to impute untyped variants as well as missing genotypes for typed variants. After imputation and quality filtering (allelic-r^2^ < 0.3 and MAF < 1%), 11,925,303 variants were available for analysis.

### Covariate identification and adjustment

Histologic characteristics and principal components derived from the SNP correlation matrix were evaluated for their association with global transcript abundance using linear or logistic regression. Principal components derived from the SNP correlation matrix exhibited minimal association (minimum P-value = 4E-06), while the percentage of lymphocytic and epithelial cells present were both found to be highly associated with global transcript abundance. The latter two characteristics were included as covariates. In addition, to account for latent sources of variation in transcript abundance, we included thirteen principal components estimated from the gene expression matrix as covariates in the eQTL analysis, each explaining >1% of variation and cumulatively explaining 43% of the total variation.

### PrCa-risk regions and loci

PrCa-risk regions were defined as the physical region (+/- 100 Kb) surrounding the PrCa-risk variant. When PrCa-risk variants were in close proximity and thus overlapping risk regions, a single risk region was defined including multiple PrCa-risk variants with physical bounds defined as +/- 100 Kb from the outermost PrCa-risk variants. Risk loci consisted of a PrCa-risk variant and all SNPs in LD (r^2^≥0.5) within its risk region (**S2 Table**). PrCa-risk variants in LD (r^2^≥0.5) were treated as a single risk locus with multiple PrCa-risk variants during analysis.

### PrCa-risk variant eQTL: Primary analysis

All variants in each PrCa-risk locus were analyzed. For a given variant, all expressed genes within 1.1 Mb were considered eligible for eQTL analysis. We evaluated eQTL associations under an additive genetic model using linear regression methods implemented in the MatrixEQTL R package, adjusting for the covariates listed above.[60] To facilitate discovery, we adopted a gene-centric false discovery rate approach to Type I error control instead of the more conservative Bonferroni correction. Specifically, gene-wise significance was determined by first applying a Bonferroni adjustment to eQTL association results for each tested gene (i.e., accounting for the number of tests performed for each gene), then calculating false discovery rate (FDR) q-values for the set of minimum per-gene Bonferroni-adjusted p-values. We declared genes with a FDR q-value ≤ 1% as “gene-wise” significant. Gene-wise significance thresholds were calculated for each gene class (i.e., protein-coding, lncRNA, other RNA) separately. The other RNA gene class included antisense RNA, miRNA, processed transcripts, pseudogenes, snoRNA, intronic, and overlapping sense RNA transcripts.

### Gene-based eQTL: Second stage analysis

To identify potential additional nearby variants affecting gene expression, a second-stage gene-based eQTL analysis (**Fig 1A**) was performed for all variants surrounding significant target genes (+/- 1.1 Mb from the transcription start site and transcription end site) identified in the primary analysis. Variant-level FDR q-values were calculated considering eQTL association p-values across all significant target genes. Regional association plots were generated using locally written R functions with empirical LD estimates obtained using PLINK v1.9 (https://www.cog-genomics.org/plink2).

It is likely that some significant eQTL signals will reflect normal, prostate tissue-specific signals. To aid interpretation and focus on genes of probable relevance to PrCa, significant target genes were classified according to the LD between the PrCa-risk variant and the peak eQTL variant considering all variants analyzed in both the primary- and second-stage. Genes with high LD (r^2^ ≥ 0.5) between the PrCa-risk variant and the peak eQTL variant were considered the most likely to be related to PrCa-risk and classified as Group 1. Those with lower LD between the PrCa-risk variant and the peak eQTL variant (r^2^ in [0.2–0.5) or r^2^ <0.2) were classified as Group 2 and Group 3, respectively.

### Functional annotation of regulatory variants

Publicaly available data were used to annotate the peak eQTL SNPs for several genes. PrCa-specific enhancers were described in Hazelett et al.[12] RegulomeDB scores (http://www.regulomedb.org/) were used to identify SNPs overlapping known and predicted regulatory elements.[42] Transcription factor binding sites were obtained from http://factorbook.org.[57] Chromatin states for normal prostate epithelial cells (PrEC) and a cancerous PrCa cell line (PC3) were obtained from Taberlay et al.[41]

## Supporting information

S1 Table Novel PrCa-risk variants included in primary analysis. (XLSX)

S2 Table PrCa-risk loci. (XLSX)

S3 Table Significant eQTL variants for *TAX1BP1*. (XLSX)

S4 Table Significant eQTL variants for *SKIL*. (XLSX)

S5 Table Significant eQTL variants for *RP11-351C8*. (XLSX)

S6 Table Gene group classification. (XLSX)

## Abbreviations

eQTL: expression quantitative trait loci
GWAS: genome-wide association study
H&E: hematoxylin and eosin
LD: linkage disequilibrium
lncRNA: long non-coding RNA
MAF: minor allele frequency
ncRNA: non-coding RNA
PrCa: prostate cancer
SNP: single nucleotide polymorphism
TCGA: The Cancer Genome Atlas
